# Halibut mitochondrial genomes contain extensive heteroplasmic tandem repeat arrays involved in DNA recombination

**DOI:** 10.1186/1471-2164-9-10

**Published:** 2008-01-11

**Authors:** Kenneth A Mjelle, Bård O Karlsen, Tor E Jørgensen, Truls Moum, Steinar D Johansen

**Affiliations:** 1 Department of Fisheries and Natural Sciences, Bodø University College, N-8049 Bodø, Norway; 2 Department of Molecular Biotechnology, Institute of Medical Biology, University of Tromsø, N-9037 Tromsø, Norway

## Abstract

**Background:**

Halibuts are commercially important flatfish species confined to the North Pacific and North Atlantic Oceans. We have determined the complete mitochondrial genome sequences of four specimens each of Atlantic halibut (*Hippoglossus hippoglossus*), Pacific halibut (*Hippoglossus stenolepis*) and Greenland halibut (*Reinhardtius hippoglossoides*), and assessed the nucleotide variability within and between species.

**Results:**

About 100 variable positions were identified within the four specimens in each halibut species, with the control regions as the most variable parts of the genomes (10 times that of the mitochondrial ribosomal DNA). Due to tandem repeat arrays, the control regions have unusually large sizes compared to most vertebrate mtDNAs. The arrays are highly heteroplasmic in size and consist mainly of different variants of a 61-bp motif. Halibut mitochondrial genomes lacking arrays were also detected.

**Conclusion:**

The complexity, distribution, and biological role of the heteroplasmic tandem repeat arrays in halibut mitochondrial control regions are discussed. We conclude that the most plausible explanation for array maintenance includes both the slipped-strand mispairing and DNA recombination mechanisms.

## Background

Halibuts (family Pleuronectidae) represent the largest of the flatfish species. Whereas Atlantic halibut (*Hippoglossus hippoglossus*) and Pacific halibut (*Hippoglossus stenolepis*) are endemic species confined to the North Atlantic and North Pacific Oceans, respectively, the Greenland halibut (*Reinhardtius hippoglossoides*) has an Arctic-boreal distribution in both the Atlantic and Pacific Oceans. All three species are commercially important flatfishes with extensive annual catch volumes, and the Atlantic halibut has further become increasingly popular in North European aquaculture [1]. Phylogenetic analysis based on partial mitochondrial DNA (mtDNA) sequences supports a sister taxa affiliation of the *Hippoglossus *and *Reinhardtius *halibuts among the Pleuronectidae [2].

Genetic markers have been developed to investigate and assess genetic issues within e.g. taxonomy, systematics, conservation biology, population structuring, or breeding programs. Mitochondrial DNA (mtDNA) has become one of the most popular genetic markers [3,4] due to its small size and stable organization, its simple inheritance pattern (maternal without apparent DNA recombination), high copy number, and elevated mutation rate compared to single-copy nuclear DNA. Vertebrate mtDNA is usually less than 17 kb in size with a plasmid-like organization, encoding only 37 gene products (13 protein-coding genes, 22 transfer RNA genes, and 2 ribosomal RNA genes) as well as a main control region (CR) containing transcriptional promoters, at least one of the replication origins as well as the displacement loop (D-loop) [5].

Mitogenomics has been developed to increase the resolution of mtDNA markers by including the complete mitochondrial genome sequence in the analyses. Recently, several genetic issues in bony fishes have been successfully investigated and resolved by mitogenomic analyses, e.g. higher-order taxonomy [6,7], within-family taxonomy [8,9], within-genus taxonomy [10,11], and intraspecific variability among geographically separated populations [11,12]. However, vertebrate mtDNA has some limitations and possible shortcomings as a molecular marker that are important to be aware of, and to further investigate [13,14]. Occasional biparental inheritance has been reported, which challenges the clonal maternal nature of vertebrate mtDNA, and some of the best-known examples are found in human and mice [15,16]. Mitochondrial DNA recombination, sometimes recognizable as a consequence of biparental inheritance, appears more frequently than originally assumed but is still a rare event in vertebrates [17]. Here, heteroplasmic tandem repeat (HTR) arrays in the CR may change due to DNA recombination, with some notable examples reported from bony fishes [18-20]. Finally, mtDNA is not always a strictly neutral marker, and both direct and indirect selection has been noted [13]. ATPase6 gene variation in humans [21] and the inherited bacterial symbionts in arthropods [22] represent fascinating examples of mtDNA selection.

In the present study we have assessed the nucleotide variability in halibut mitochondrial genomes within and between species. The complete mitochondrial genome sequences from four individuals each of the Atlantic-, Pacific-, and Greenland halibuts were determined and analysed. A complex organized HTR array in the mitochondrial CR was discovered and investigated in further detail. These composite arrays provide new evidence of DNA recombination in vertebrate mitochondria.

## Results

### Gene content and organization of halibut mitochondrial genomes

**Figure 1:**
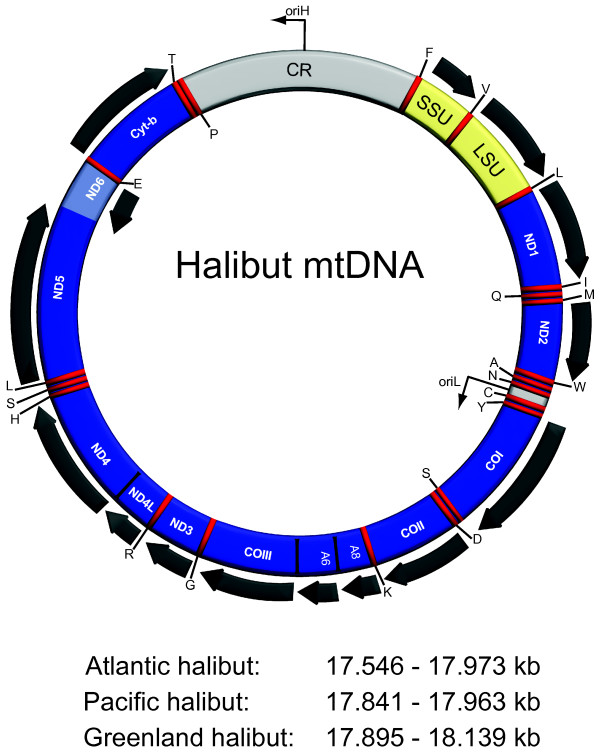
**Gene content and organization of halibut mitochondrial genomes**. Circular gene map representing the mtDNAs of Atlantic-, Pacific-, and Greenland halibuts. All genes, except ND6 and eight of the transfer RNA genes (indicated by the standard one-letter symbols for amino acids), are encoded by the H-strand. Protein genes and ribosomal RNA genes are indicated by blue and yellow boxes, respectively. The tRNA genes are indicated by red bars, and the control regions in grey boxes. Abbreviations: SSU and LSU, mitochondrial small- and large-subunit ribosomal RNA genes; ND1-6, NADH dehydrogenase subunit 1 to 6; COI-III, cytochrome c oxidase subunit I to III; A6 and A8, ATPase subunit 6 and 8; Cyt b, cytochrome b; oriH and oriL, origin of H-strand and L-strand replication; CR, control region containing the D-loop. The mitochondrial genome sizes of the 12 sequenced specimens, representing three different halibut species, are indicated below the map.

The complete mitochondrial genome sequences were determined for four individuals each of Atlantic halibut (*H. hippoglossus*), Pacific halibut (*H. stenolepsis*), and Greenland halibut (*R. hippoglossoides*) (Table 1). The circular mtDNAs were identical in gene content (13 protein coding genes, 2 ribosomal RNA genes, and 22 transfer RNA genes) and organization compared to most vertebrates, but varied in size between 17.546 kb and 18.139 kb (Figure 1; Table 1), which are about 1 kb larger than most bony fish mitochondrial genomes [23]. However, these sizes are not absolute since HTRs are observed within the mitochondrial CR (see below). The GC contents of the mitochondrial genomes were 46.1%, 45.7%, and 45.1% for Atlantic-, Pacific, and Greenland halibuts, respectively. These values are similar to most sequenced bony fish mitochondrial genomes. Furthermore, the codon usage was found to be very similar among the three halibut species investigated and with general discrimination against G at the third nucleotide position.

**Table 1:** Key features of halibut specimens and complete mtDNA sequences

**Name**	**Specimen**	**Location**	**Size (bp)**	**Acc. no**.
*Hippoglossus hippoglossus *(Atlantic halibut)	Hh-1	Northern Norway, hatchery (Bodø)	17546	AM749122
*H. hippoglossus*	Hh-2	Northern Norway, hatchery (Bodø)	17619	AM749123
*H. hippoglossus*	Hh-3	Northern Norway, wild (Bodø)	17973	AM749124
*H. hippoglossus*	Hh-4	Southern Norway, aquarium (Risør)	17729	AM749125
*H. stenolepis *(Pacific halibut)	Hs-1	Alaska, wild (Cook Inlet)	17841	AM749126
*H. stenolepis*	Hs-2	Canada, wild (Hecate Strait)	17841	AM749127
*H. stenolepis*	Hs-3	Canada, wild (Hecate Strait)	17963	AM749128
*H. stenolepis*	Hs-4	Canada, wild (Hecate Strait)	17902	AM749129
*Reinhardtius hippoglossoides *(Greenland halibut)	Rh-1	Northern Norway, wild (Røst)	18017	AM749130
*R. hippoglossoides*	Rh-2	Northern Norway, wild (Røst)	18139	AM749131
*R. hippoglossoides*	Rh-3	Northern Norway, wild (Røst)	17895	AM749132
*R. hippoglossoides*	Rh-4	Northern Norway, wild (Røst)	18078	AM749133
*Verasper variegatus *(Spotted halibut)			17273	DQ403797
*V. moseri *(Barfin flounder)			17588	EF025506

### The mitochondrial control regions contain heteroplasmic tandem repeat arrays

Intergenic regions are practically lacking in halibut mtDNAs, except for the short spacer between the tRNA-Asn and tRNA-Cys genes that contains the origin of light-strand (oriL) replication, and the major CR (contains the D-loop) located between the tRNA-Pro and tRNA-Phe genes (Figure 1). Whereas the former region is completely conserved in sequence between the 12 analysed halibut specimens, the latter is more variable and contains control elements like the origin of heavy-strand (oriH) replication and the transcriptional promoters.

**Figure 2:**
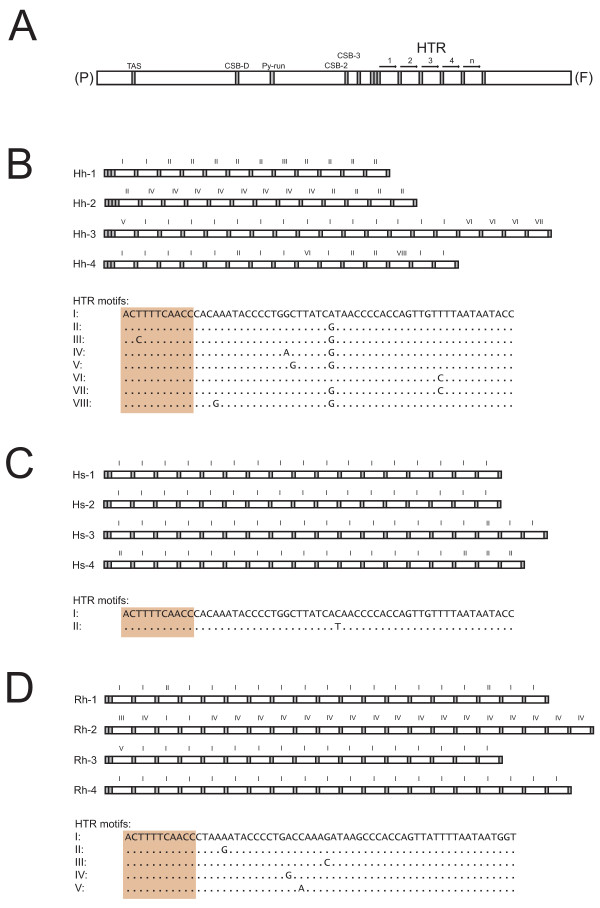
**Organization of tandem repeat arrays located within the control region of halibut mtDNAs**. (**A**) Schematic organization of the control region (CR) representing all analysed halibut species and specimens. CR is located between the tRNA genes Pro (P) and Phe (F), and contains the highly conserved termination association sequence (TAS: TACATGTATAA), conserved sequence box D (CSB-D: CCTGGCATTTGGTTCC), pyrimidine sequence run (Py-run: TTCTCTTTTTTTTTTTCCTTTC), and the two conserved sequence boxes associated with oriH (CSB-2: AAACCCCCCTACCCCCC, and CSB-2: TGAAAACCCCCCGGAAACA). The heteroplasmic tandem repeat (HTR) array is located between CSB-3 and tRNA-F gene. (**B**) Detailed view of the HTR array in cloned and sequenced CR from the Atlantic halibut (*H. hippoglossus*) specimens. The 61-bp HTR motif was found in 12, 13, 19, and 15 copies in Hh-1, Hh-2, Hh-3, and Hh-4, respectively. The HTR motif starts with an 11-bp submotif (boxed), also found freestanding flanking the 61-bp array motif. Eight different variants of the HTR motif (I-VIII) were noted in Atlantic halibut. Hh-2 has one additional freestanding 11-bp motifs compared to the other specimens. (**C**) HTR array in Pacific halibut (*H. stenolepsis*) specimens Hs-1 to Hs-4. Two different variants of the motif were found. (**D**) HTR array in Greenland halibut (*R. hippoglossoides*) specimens Rh-1 to Rh-4. Five different variants of the motif were found.

A schematic presentation of the CR organization is shown in Figure 2A. All specimens contain extensive direct repeat arrays located between the conserved sequence box (CSB) 3 and the tRNA-Phe gene. These arrays were similar among the halibut species investigated, and consist of a free-standing 11-bp motif flanking each side of the array as well as variable numbers of a 61-bp motif in between. In Atlantic halibut the investigated specimens Hh-1, Hh-2, Hh-3, and Hh-4 contain 12, 13, 19, and 15 copies, respectively, of the 61-bp motif in a plasmid cloned representative of the CRs (Figure 2B). Interestingly, eight different variants of the 61-bp motifs were identified (HTR motifs I to VIII) with a distinct, but scattered distribution pattern within and between specimens (Figure 2B). Similar patterns and distributions were observed both for the Pacific halibut (17–19 HTR copies; Figure 2C) and Greenland halibut (17–21 HTR copies; Figure 2D), but with less complexity compared to the Atlantic halibut.

**Figure 3:**
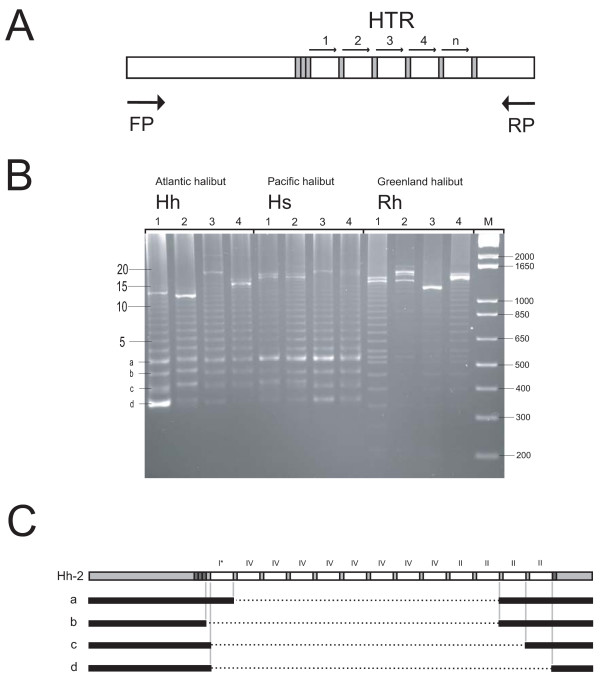
**Heteoplasmic features of HTR arrays in halibut mtDNA**. (**A**) Schematic map of the analysed HTR and flanking regions. The HTR arrays were amplified using a forward primer (FP) and a reverse primer (RP). Here, primer sets L16293/H17347 and L16376/H17369 were used on Atlantic/Pacific halibuts and Greenland halibut, respectively. (**B**) Separation of amplified products in a 2.5% agarose gel. M, the size marker 1 Kb Plus DNA Ladder from Invitrogen (right). Number of repeat motifs is indicated (left). The four smallest amplified fragments from Hh-2 (marked a, b, c, and d) were eluted from the gel and further sequence analysed. (**C**) Summary of sequence analyses of fragment a-d from Hh-2. Note that fragment a contains three repeats of the 61-bp motif (I*, II, II), fragment b contains two repeat motifs (II, II), fragment c contains a single motif (II), and fragment d lacks a complete motif. Motif I* is not present in the plasmid cloned array of Hh-2 (Figure 2) and may represent site heteroplasmy at array motif position 33.

A PCR-amplification and DNA sequencing approach was included to assess the heteroplasmic patterns of the HTR-motif arrays. The HTR region and some flanking sequences were amplified from DNA isolated from all 12 specimens (Figure 3A) and subsequently separated by agarose electrophoresis (Figure 3B). Extensive heteroplasmic features were observed for all 12 specimens, with the most common copy numbers of the 61-bp repeat of about 15–20. The four smallest amplified fragments (named a-d in Figure 3B) from one specimen of Atlantic halibut (Hh-2) were eluted from the gel, cloned into a plasmid vector, and subsequently DNA sequenced. The results are summarized in Figure 3C and confirm that the fragments present in the agarose gel as ladder patterns differs in size by one 61-bp motif. Surprisingly, the smallest fragment (fragment d) lacks a complete 61-bp motif and only contains a few copies of the free-standing 11-bp motif.

### Distribution of sequence variation within the halibut mitochondrial genomes

**Figure 4:**
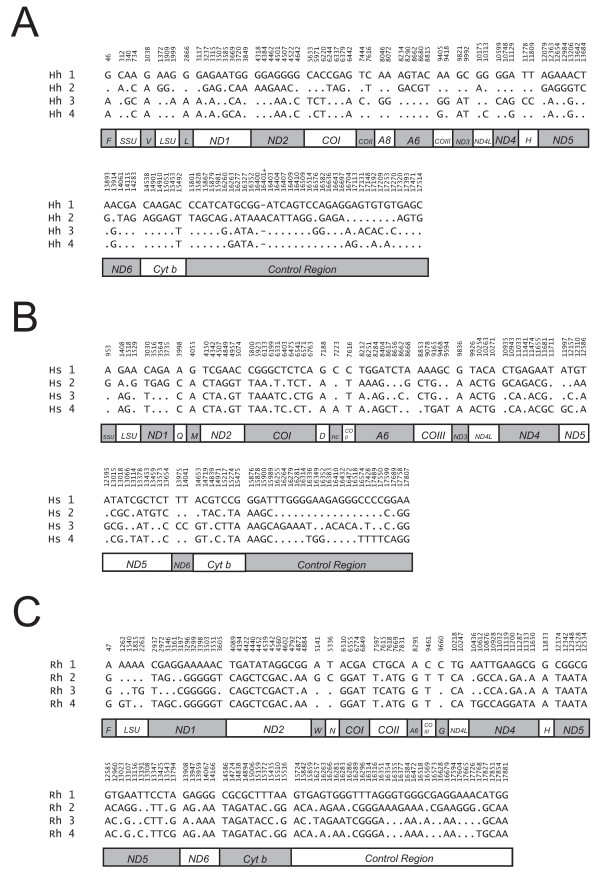
**Variability of halibut mitochondrial genomes**. (**A**) Distribution of variable sites in Atlantic halibut mtDNA numbered according to the sequence of specimen Hh-1 (AM749122; Table 1). The variable sites were aligned to that of Hh-1. Identical sites are indicated by dots and deletions by dashes. Abbreviations are according to the legend to Figure 1. The first and last three 61-bp motifs in the CR heteroplasmic array were included in the alignment. (**B**) Variable sites in Pacific halibut mtDNA numbered according to the sequence of specimen Hs-1 (AM749126; Table 1). (**C**) Variable sites in Greenland halibut mtDNA numbered according to the sequence of specimen Rh-1 (AM749130; Table 1).

Nucleotide substitutions and deletions were assessed by comparing the complete mtDNA sequence of the 4 specimens of each halibut species. The total numbers of variable sites identified were 105, 103, and 119 in Atlantic-, Pacific-, and Greenland halibuts, respectively. The variable sites include all protein coding and ribosomal RNA genes, the CR, and nine of the 22 transfer RNA genes (Figure 4). Transition substitutions at third codon positions of protein coding genes were the most common changes, and nucleotide deletions were only observed at one site in the Atlantic halibut CR.

In order to further evaluate the distribution of sequence variation among the halibut mitochondrial genes, both within and between species, substitution versus nucleotide position was estimated for the different gene regions. This ratio was then divided by the ratio obtained for the mitochondrial ribosomal DNA (mt-rDNA; mtSSU + mtLSU; 2665 bp) in order to estimate a relative number of the mitochondrial gene region variation that could be compared between different species and datasets. Despite the fact that numbers of specimens are too low to perform statistics, some general trends were seen (Table 2). First, protein coding genes were about 3–4 times more variable than mt-rDNA both within and between species. The cytochrome c oxydase (CO) subunits were slightly more conserved than the other mitochondrial encoded subunits. Second, the tRNA gene pool (ca 1550 bp) has a similar sequence variation rate as the mt-rDNA. Finally, whereas the CR possesses a between-species sequence variation similar to that of most of the protein genes, it was clearly elevated within the halibut species. In fact, the CRs in Atlantic-, Pacific, and Greenland halibuts were about 10 times more variable per site than the corresponding mt-rDNAs.

**Table 2:** Estimates of gene specific variation related to the mitochondrial ribosomal DNA gene region

**Gene ^1^**	**Halibuts; within-species^2^**		**Halibuts; between-species^3^**		***Theragra*; within-species^4^**	
	**Observed**	**Relative**	**Observed**	**Relative**	**Observed**	**Relative**
ND1	0.0079	4.4	0.186	4.0	0.0226	4.6
ND2	0.0080	4.4	0.216	4.6	0.0163	3.3
ND3	0.0029	1.6	0.189	4.0	0.0172	3.5
ND4L	0.0090	5.0	0.128	2.7	0.0034	0.7
ND4	0.0051	2.8	0.199	4.2	0.0167	3.4
ND5	0.0067	3.7	0.209	4.4	0.0207	4.2
N6	0.0077	4.3	0.201	4.3	0.0172	3.5
COI	0.0045	2.5	0.151	3.2	0.0109	2.2
COII	0.0039	2.2	0.152	3.2	0.0043	0.9
COIII	0.0034	1.9	0.144	3.1	0.0140	2.9
A6	0.0068	3.8	0.198	4.2	0.0161	3.3
A8	0.0040	2.2	0.137	2.9	0.0119	2.4
Cyt B	0.0067	3.7	0.193	4.1	0.0158	3.2
CR ^5^	0.0211	11.7	0.220	4.7	0.0160	3.3
tRNA	0.0028	1.6	0.062	1.3	0.0013	0.3
SSU	0.0014	0.8	0.042	0.9	0.0063	1.3
LSU	0.0019	1.1	0.050	1.1	0.0041	0.8
SSU + LSU	0.0018	1.0	0.047	1.0	0.0049	1.0

Notes: ^1 ^Protein genes – all gene positions except stop codons. The variable sites are mainly transition substitutions at third codon positions. CR – control region nucleotides including the first and last three motifs of tandem repeates. tRNA genes – pool of all 22 tRNA genes, ca 1550 bp; SSU + LSU – combined mitochondrial small and large ribosomal subunit RNA genes, ca 2665 bp.

^2 ^Estimated avarage values within the three halibut species (Atlantic-, Pacific-, and Greenland halibuts) based on four specimens of each. The observed variation value is number of variable sites divided on total number of nucleotides of that particular mitochondrial region. The relative variation value is the observed variation divided on the combined SSU/LSU variation.

^3 ^Estimated nucleotide variation between halibut species and includes the Atlantic halibut (Hh-1 specimen), Pacific halibut (Hs-1 specimen), Greenland halibut (Rh-1 specimen), Spotted halibut, and Barfin flounder. Key features of species are given in Table 1.

^4 ^Estimated nucleotide variation within pollocks (*Theragra*; Gadidae) and is based on 12 specimens representing one single species [11, 12].

^5 ^The elevated substitution level in CR within halibut species is boxed.

**Figure 5:**
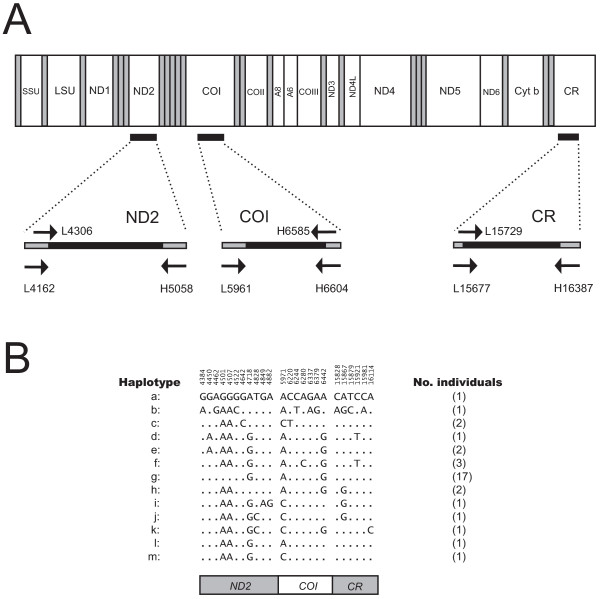
**Mitochondrial haplotype in Atlantic halibut**. (**A**) Amplification strategy of partial ND2, COI, and CR mitochondrial sequences. PCR primers and sequencing primers (Table 2) are indicated below and above the schematic line-drawings, respectively. (**B**) Summary of haplotypes detected within the ca 1770 bp sequence. Haplotype g refers to sibling or half sibling progenies from halibut hatchery.

Based on the knowledge about gene specific variability within halibut mtDNA we selected three regions with moderate to high sequence variation (parts of ND1, COI, and CR) for an extended study of Atlantic halibut. Thirty additional specimens (15 wild caught and 15 farmed progenies) from a halibut hatchery were subjected to mtDNA PCR analysis (Figure 5A) and subsequently DNA sequencing. Here, a 1770 bp region was analysed and compared, and the results are summarized in Figure 5B. When including the 4 completely sequenced specimens (Hh-1 to Hh-4), 24 variable sites were detected resulting in 13 distinct haplotypes (Haplotypes a-m). Interestingly, 15 specimens possess an identical haplotype (Haplotype g; Figure 5B), all corresponding to the farmed progenies that probably reflect siblings, or half siblings. This result supports the potential of mtDNA as molecular marker in breeding programs, stock assessments, or population studies.

## Discussion

We have sequenced and compared the complete mitochondrial genome sequences of 4 individuals each of the flatfish species Atlantic halibut, Pacific halibut, and Greenland halibut, all related members of the family Pleuronectidae. The mitochondrial genomes were similar to most other bony fish species, except for an unusual large and complex CR located between the tRNA-Pro and tRNA-Phe genes. Halibut CR contains a HTR array of a 61-bp motif, most frequently present in 15–20 copies of each individual.

The within-species variation in mtDNA includes only about 20–100 sequence positions between the individuals. These numbers correlates well with those observed among 12 individuals of *Theragra *pollocks [11,12]. The variable sites are not equally distributed along the mitochondrial genome sequence, with the structural RNA genes as the most conserved sequence regions (Table 2). The latter observation is best explained by the complex structural constrains of their corresponding tRNA and rRNAs due to secondary and tertiary RNA:RNA interactions, as well as RNA:protein interactions. Interestingly, the structure determination of the vertebrate mitochondrial ribosome explains some of the dramatic reduction in size of the mitochondrial rRNAs, which leaves almost exclusively the highly conserved regions involved in ribosome function and ribosomal protein binding [24].

The elevated within-species sequence variation in CR observed in all three halibut species (Table 2) appears unique compared to other investigated fish mtDNA genomes. The only fish species were complete mtDNA sequences have been recovered from multiple specimens (12 individuals) is the *Theragra *pollocks [11,12]. Intraspecific sequence variability estimates were similar to that of the halibut species, but with a notable exception of the CR. The *Theragra *CR showed variability similar to that of the protein coding genes, an observation significantly different from that of halibuts (Table 2). The variable sites in halibuts are almost exclusively located in the extended termination associated sequence (ETAS) and CSB regions located at the 5' end and 3' end of the CR, respectively. What molecular processes that causes this elevated sequence variability is currently not known, but DNA recombination events at HTR arrays (see below) are likely to be involved.

HTRs in mitochondrial CR are widespread, but scattered among vertebrates [18]. Five different locations within the mitochondrial CR have been noted to harbour HTRs [25]. Whereas the RS1 and RS2 sites are located at the CR 5' end in proximity to the termination association sequence, RS3 to RS5 are located close to the oriH replication at the 3' end of the CR. Thus, the presence of HTR in CR is probably associated with the DNA replication processes in vertebrate mtDNA [13]. The complexity of HTR motifs vary greatly among different vertebrates, from simple di- and tetra-nucleotide microsatellites to motifs more than 150 bp in length and at high copy numbers [26-28].

The halibut 61-bp motif HTR array is located at site RS5 between CSB-3 and the 3' end of CR (Figure 2A), and differs from the RS1 HTR arrays seen in e.g. Atlantic cod and Asian arowana that consist of only 2–6 copies of approximately 40-bp motifs [28-30]. The RS5 HTR is a conserved feature among the Pleuronectidae where a ca 60-bp motif array is present in e.g. Spotted halibut (*Verasper variegatus*; DQ403797), Barfin flounder (*V. moseri*; EF025506), Winter flounder (*Pseudopleuronectes americanus*), Yellowtail flounder (*Limanda ferruginea*), and American plaice (*Hippologlossoides platessoides*) [31], in addition to the three halibut species investigated in this study. Interestingly, RS5 direct repeats are also noted in Soleidae, but these appear unrelated in sequence to the Pleuronectidae HTRs and do not create heteroplasmy in mtDNA [32]. Partial sequencing of the mitochondrial CR in European flounder (*Platichthys flesus*) identified a different repeat motif at RS1 [19,33]. This 19-bp motif was involved in extensive heteroplasmy identified in a study including 168 individuals [19]. Interestingly, two different types of repeat motifs were noted among the 18 individuals studied in more detail, and one of these contains a compound array consisting of both motif types. Our finding of multiple types of HTR motifs in Atlantic-, Pacific-, and Greenland halibuts represents an extended support of the observation in European flounder. Errors during mtDNA replication (e.g. slipped strand mispairing) [34] cannot fully explain the halibut length heteroplasmy since repeat motifs in arrays of most individuals are not identical (Figure 2). Furthermore, technically generated mutations in the sequences, as well as the possibility that an ancestral sequence variant that contained all motif variants, are both highly unlikely explanations since the same type of motifs appear in more than one species and that eight different types were present among the four individuals of Atlantic halibut. Thus, we strongly favour DNA recombination as the most plausible mechanism, a conclusion supporting the findings of Hoarau and co-workers in European flounder mtDNA [19].

Is there a biological role of the mitochondrial HTR arrays in halibuts? The facts that repeat motifs are highly conserved in sequence both between individuals and between Pleuronectidae species (Figure 2) indicate a functional role in the mitochondria. However, mitochondrial genomes lacking the motif, or with only a single copy present (Figure 3), favour no essential role of the array or the motif sequences. The deletion variant (fragment d in Figure 3C) may represent a dead-end of array heteroplasmy unless the HTR motif is reintroduced by DNA recombination. Interestingly, the deleted region is flanked by identical copies of the 11-bp motif and thus probably is generated by a slipped strand mispairing-like process [34], similar to that reported in mitochondria associated with some human diseases [35]. The HTR arrays in halibuts are located between the putative promoter region (3' end of CR) and oriH, and HTRs in RS5 have been functionally linked to the initiation of mtDNA replication [27]. A role of stable secondary structures of nucleotide repeats nucleotides has been suggested. Such putative structures might act at the RNA or DNA levels [36], but at present no experimental biochemical evidence has been provided to support this notion in mitochondria. To further elucidate the molecular evolution and biological roles of HTR arrays in halibut mitochondrial genomes, investigations of the distribution and variation of arrays among different tissues and at different developmental stages should be performed. Studying array variability of mother and progeny would be of particular interest in order to identify possible DNA recombination events. The well studied example of similar RS5 arrays in mitochondria of European rabbits provides an interesting model system for such analyses [27,37-39].

## Conclusion

Unusual molecular features of halibut mitochondrial genomes are located in the control region. Extensive size heteroplasmy was detected in Atlantic-, Pacific-, and Greenland halibut mitochondrial control regions. Heteroplasmic tandem repeat arrays contain different variants of a 61-bp motif in compound organization. We conclude that the most plausible explanation for array maintenance includes both slipped-strand mispairing and DNA recombination mechanisms.

## Methods

### Fish samples and DNA extraction

Key-features of fish samples and mitochondrial DNA sequences used in this study are listed in Table 1. Of the 30 additional Atlantic halibut specimens obtained from a halibut hatchery at Bodø University College, 15 were wild caught (Northern Norway) and 15 were farmed progenies from the hatchery. DNA was extracted from muscle tissue and fin clip by using the High Pure PCR Template Preparation Kit (Roche).

### PCR amplification, cloning, DNA sequencing, and data analysis

Specific primer sets consisting of one heavy (H) and one light (L) strand primer (Additional file 1) were used to amplify the complete halibut mitochondrial genomes in five overlapping fragments (L466/H3978, L3851/H7461, L7109/H10004, L9620/H13706, and L12991/H530). In general, the PCR reactions were performed with the following cycling parameters: 94°C initial denaturation for 3 min, 15 cycles with 94°C denaturation for 60 sec, 48°C annealing for 60 sec, 72°C elongation for 4 min. Then, 15 cycles with 94°C denaturation for 60 sec, 53°C annealing for 60 sec, 72°C elongation for 4 min and finally 72°C for 10 min. Products were run on agarose gels containing ethidium bromide, and bands were excised and purified essentially as previously described [11]. When appropriate, PCR products were inserted into the pCR4-TOPO vector (Invitrogen) and transformed in *E. coli *competent cells. PCR products were sequenced on both strands by using the BigDye version 3.1 kit (Applied Biosystems) with the same primers as in the PCR and internal primers (Additional file 1). The sequencing products were analysed on an ABI genetic analyser (Applied Biosystems). In general, computer analyses of DNA sequences were performed using software package programs from DNASTAR Inc.

## Authors' contributions

KAM, TEJ, and BOK organized the sequencing of the mitochondrial genomes. SDJ, BOK, KAM, TM, and TEJ contributed to mtDNA sequence analyses. SDJ directed the research in collaboration with BOK and TM. SDJ wrote the paper in collaboration with BOK. All authors read and approved the final manuscript version.

## Supplementary Material

Additional file 1 PCR and DNA sequencing primers. Sequence information and location of DNA primers used in PCR and sequencing reactions Click here for file
