# Supplementary material for: Halibut mitochondrial genomes contain extensive heteroplasmic tandem repeat arrays involved in DNA recombination
Source: BMC Genomics. 2008 Jan 11;9:10. doi: 10.1186/1471-2164-9-10 (PMC2248175; doi:10.1186/1471-2164-9-10)
Supplement: Additional file 1 — PCR and DNA sequencing primers. Sequence information and location of DNA primers used in PCR and sequencing reactions [file 1471-2164-9-10-S1.doc]

**Additional file 1** PCR and DNA sequencing primers

___________________________________________________________________________

**Primer Sequence (5’, 3’) Primer Sequence (5’, 3’)**

H30 cccatcttaacatcttca L8825 aagcctctacctacaagaaa

L466 gtagctctactcatcctgaa H8833 gggtgtgcttgatgggccat

H530 atatggagtgtatagtgcaa L9620 tactgatgaggatcttaatc

L1251 cgcaagggaaagctgaaa H9950 gctgtagctcaggtgaacgtc

L1651 gatcatattcaaataaggaca H10004 gacctccttgcattcattcg

H1839 gtccgttccgatttacac H10066 aaccatggwtttttgagccgaaat

L2165 taaatgaagacctgtatgaa L10173 taacaggcctggcattccac

H2628 tccggtctgaactcagatcacgta L11368 attgcacacgggcttacttc

L2643 gtcctacgtgatctgagttc H11573 caccggttaatgccagagtt

L2957 ttgctagccgtagcattcct L11817 aaacactagattgtgattct

L3851 cgctggtctcccaccacagct H11935 ctaagaccaacggatgagct

H3978 aagtggtgtagaggaagcac L12582 catggcttggaatagcaacaaac

L4162 actggctcctcgcttgaata H12728 agtagggcagataccggtgtag

L4306 caagcactaccaacgcttga L12991 caaccccaactagcatttcttc

H4731 cattgacggtggtcgctttgt H13706 ggtaggttagaggatgcaatgg

L4771 ccctcgccatctcgtgaaca L14102 acaccattaccgaccaactacc

H5058 gagaagaagggtggttgctg H14394 ttgtagttgaataacaacgg

L5622 agtctgcttattcgggcaga L14409 ccaccgttgttattcaact

L5961 caatcttctcacttcaccttgc L15677 ctacccctaactcccaaagc

H6198 aaagaatcagaataggtgtt L15729 cataaatgtacaatgaagg

H6585 cagaacaatctcgagagagg H16203 gagaaccccttacccgctggagtgaac

H6604 actacatagtatgtgtcatgc L16293 cggagacgtttaaagggt

L7901 atcaattggtttcaagccaa L16376 acataaagggatatcatgtgc

H7178 acaattctgccttgacaagg H16387 ggatgacagaagaaatgagc

H7461 agtacaaaattcggatggatgg H17347 cttgaaataatctaaacagta

L8114 ctacccctaactcccaaagc H17540 gttgggatgatgcaagaaat

H8143 aagcttagtatcatggtcagt

**Note**. The primers are numbered relative to the position of the 3’ nucleotide in the Atlantic halibut specimen Hh-1 (AM749122). H and L refer to the heavy and light strand, respectively.
